# Translational molecular imaging and drug development in Parkinson’s disease

**DOI:** 10.1186/s13024-023-00600-z

**Published:** 2023-02-10

**Authors:** Achi Haider, Nehal H. Elghazawy, Alyaa Dawoud, Catherine Gebhard, Thomas Wichmann, Wolfgang Sippl, Marius Hoener, Ernest Arenas, Steven H. Liang

**Affiliations:** 1 Department of Radiology, Division of Nuclear Medicine and Molecular Imaging Massachusetts General Hospital and Harvard Medical School, 55 Fruit Street, Boston, MA 02114 USA; 2 Department of Radiology and Imaging Sciences, Emory University, 101 Woodruff Circle, Atlanta, GA 30322 USA; 3 Biochemistry Department, Faculty of Pharmacy and Biotechnology, German University in Cairo, Main Entrance of Al-Tagamoa Al-Khames, Cairo, 11835 Egypt; 4 Molecular Genetics Research Team (MGRT), Pharmaceutical Biology Department, Faculty of Pharmacy and Biotechnology, German University in Cairo, Main Entrance of Al-Tagamoa Al-Khames, Cairo, 11835 Egypt; 5 Department of Nuclear Medicine, University Hospital Zurich, Raemistrasse 100, 8091 Zurich, Switzerland; 6 Center for Molecular Cardiology, University of Zurich, Schlieren, Switzerland; 7 Department of Neurology/School of Medicine, Yerkes National Primate Research Center, Emory University, Atlanta, GA USA; 8 Institute of Pharmacy, Department of Medicinal Chemistry, Martin-Luther-University Halle-Wittenberg, W.-Langenbeck-Str. 4, 06120 Halle, Germany; 9 Neuroscience and Rare Diseases Discovery and Translational Area, Roche Innovation Center Basel, F. Hoffmann-La Roche, Grenzacherstrasse 124, 4070 Basel, Switzerland; 10 Karolinska Institutet, MBB, Molecular Neurobiology, Stockholm, Sweden

**Keywords:** Translational molecular imaging, Parkinson’s disease, Drug development, Dopamine, α-Synuclein, Mitochondrial dysfunction, Neuroinflammation, Neurodegeneration

## Abstract

Parkinson’s disease (PD) is a progressive neurodegenerative disorder that primarily affects elderly people and constitutes a major source of disability worldwide. Notably, the neuropathological hallmarks of PD include nigrostriatal loss and the formation of intracellular inclusion bodies containing misfolded α-synuclein protein aggregates. Cardinal motor symptoms, which include tremor, rigidity and bradykinesia, can effectively be managed with dopaminergic therapy for years following symptom onset. Nonetheless, patients ultimately develop symptoms that no longer fully respond to dopaminergic treatment. Attempts to discover disease-modifying agents have increasingly been supported by translational molecular imaging concepts, targeting the most prominent pathological hallmark of PD, α-synuclein accumulation, as well as other molecular pathways that contribute to the pathophysiology of PD. Indeed, molecular imaging modalities such as positron emission tomography (PET) and single-photon emission computed tomography (SPECT) can be leveraged to study parkinsonism not only in animal models but also in living patients. For instance, mitochondrial dysfunction can be assessed with probes that target the mitochondrial complex I (MC-I), while nigrostriatal degeneration is typically evaluated with probes designed to non-invasively quantify dopaminergic nerve loss. In addition to dopaminergic imaging, serotonin transporter and *N-*methyl-D-aspartate (NMDA) receptor probes are increasingly used as research tools to better understand the complexity of neurotransmitter dysregulation in PD. Non-invasive quantification of neuroinflammatory processes is mainly conducted by targeting the translocator protein 18 kDa (TSPO) on activated microglia using established imaging agents. Despite the overwhelming involvement of the brain and brainstem, the pathophysiology of PD is not restricted to the central nervous system (CNS). In fact, PD also affects various peripheral organs such as the heart and gastrointestinal tract – primarily via autonomic dysfunction. As such, research into peripheral biomarkers has taken advantage of cardiac autonomic denervation in PD, allowing the differential diagnosis between PD and multiple system atrophy with probes that visualize sympathetic nerve terminals in the myocardium. Further, α-synuclein has recently gained attention as a potential peripheral biomarker in PD. This review discusses breakthrough discoveries that have led to the contemporary molecular concepts of PD pathophysiology and how they can be harnessed to develop effective imaging probes and therapeutic agents. Further, we will shed light on potential future trends, thereby focusing on potential novel diagnostic tracers and disease-modifying therapeutic interventions.

## Background

Parkinson’s disease (PD) is a progressive neurodegenerative disorder that mainly affects elderly people [1]. The clinical diagnosis of PD is based on the presence of a constellation of movement abnormalities, including the presence of slowness of movement (bradykinesia), poverty of movement (akinesia), tremor at rest, and muscle stiffness (rigidity). Although the history of parkinsonism dates back to the ancient Indian medical literature [2], the first coherent description of PD-related clinical symptoms was reported in 1817 by James Parkinson in his seminal work ‘An essay on the shaking palsy’ [3, 4]. Notably, the incidence and prevalence of PD have risen rapidly in the past two decades, contributing to a mounting socioeconomic burden [5]. Along this line, epidemiological studies unveiled that the age-standardized global prevalence of PD has more than doubled from 1990–2016, thereby exhibiting the highest growth rate among all major neurological disorders [6]. While underlying causes for this disproportionate growth are not fully understood, the alerting development calls for improved diagnostic and therapeutic measures.

PD has traditionally been classified into two distinct forms, namely, the hereditary form with genetic mutations known to contribute to the pathophysiology of PD and the idiopathic form, which is essentially of unknown origin [7]. In 2004, Braak et. al. introduced a PD staging metric comprising six neuropathological phenotypes that included both, presymptomatic and symptomatic disease stages [8]. Of note, while initial pathological stages included the formation of intracellular inclusion bodies (mainly aggregates of misfolded α-synuclein) in neurons of asymptomatic individuals, more advanced disease stages were characterized by degeneration of dopaminergic neurons in the substantia nigra pars compacta. Many of the prototypical parkinsonian motor signs (see above) are due to the loss of dopamine in the striatum, the primary recipient of nigral efferents. The brain has a remarkable ability to compensate for the loss of nigrostriatal fibers: It is estimated that up to 50–80% of dopaminergic cells in the nigrostriatal system can be lost before motor symptoms manifest [9–11]. In later stages, neurodegeneration is observed in the neocortex and other brain regions, leading to additional (non-dopaminergic) symptoms [8, 12].

**Fig. 1:**
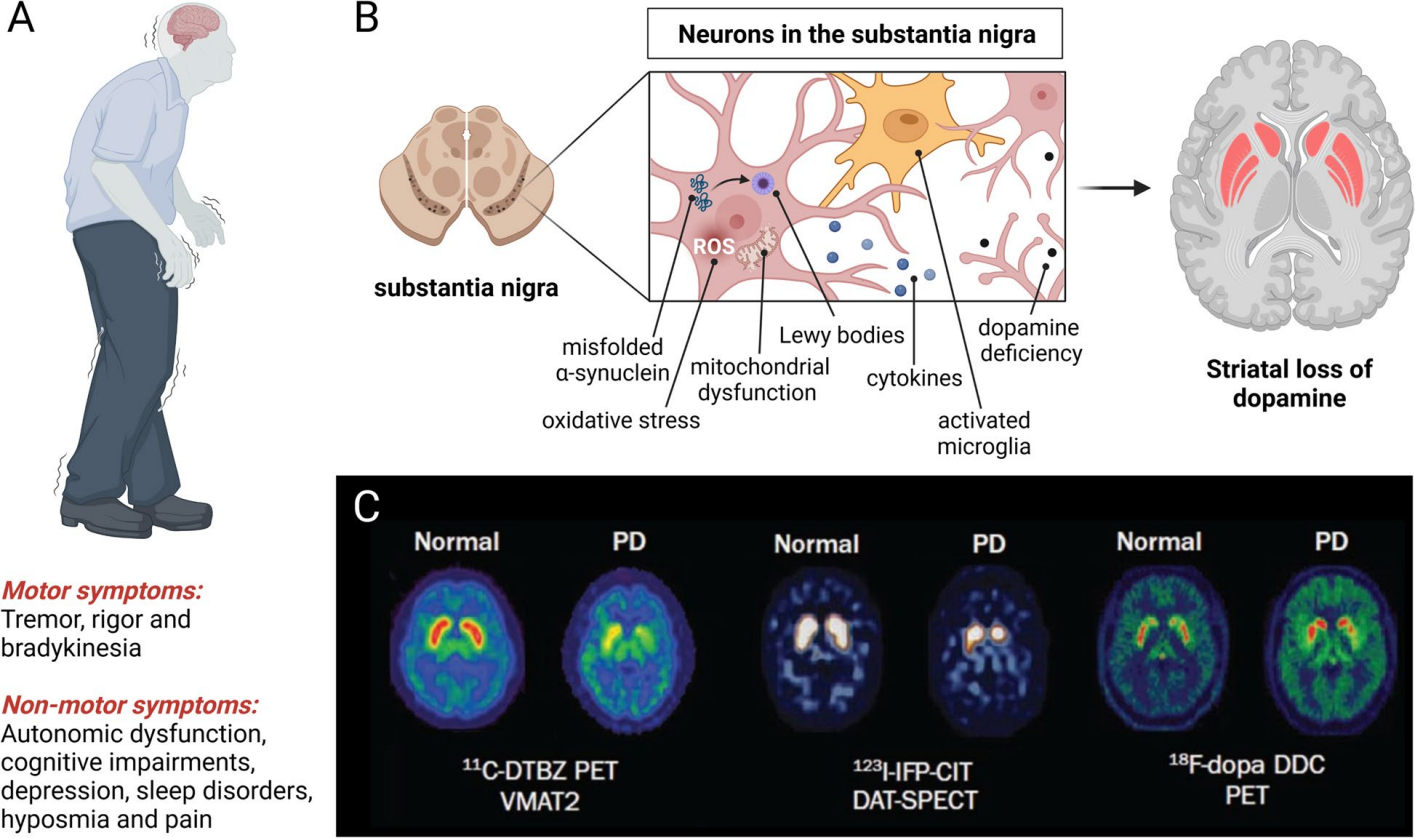
Clinical presentation, pathophysiology and diagnostic imaging in Parkinson’s disease (PD). **A** Cardinal motor symptoms and non-motor features of PD. **B** Pathological hallmarks of PD include aggregates of misfolded α-synuclein (Lewy bodies), mitochondrial dysfunction & oxidative stress, infiltration of immune cells and persistent activation of microglia, as well as an increased release of cytokines. The pathophysiology of PD prompts neurodegeneration and dopamine deficiency, which is particularly pronounced in the mammalian striatum. **C** Translational molecular imaging with positron emission tomography (PET) and single-photon emission computed tomography (SPECT), visualizing striatal degeneration in PD non-invasively. DAT, dopamine transporter.; DDC, DOPA decarboxylase; VMAT2, vesicular monoamine transporter 2. Figure 1C reprinted by permission from the Royal College of Physicians (RCP): Pagano et al., [17] copyright (2016)

Although the clinical presentation of PD is dominated by motor symptoms (Fig. 1A), it has become apparent that the majority of patients also display a multitude of non-motor features [13]. Indeed, individuals with prodromal disease may first experience one or several of the following symptoms, including autonomic dysfunction, cognitive impairments, depression, sleep disorders, hyposmia and pain [10, 14–16]. Accordingly, PD constitutes a clinical syndrome that affects multiple organs.

Molecular imaging has contributed to a better understanding of a variety of molecular and cellular concepts that have been linked to PD onset and progression, including mitochondrial dysfunction [18, 19], oxidative stress [20, 21], neuroinflammation [22, 23], excitotoxicity [24], genetic mutations [25, 26], failure of protein clearance pathways and abnormal protein aggregation (Fig. 1B) [27]. Amyloid-based imaging has been suggested to aid the clinical evaluation of PD-associated forms of dementia, in particular, to assess whether cognitive decline is occurring in the setting of abnormal Aβ accumulation [28]. With the development of improved sensitivity of detection modalities, PET now allows the assessment of reactive oxygen species (ROS) in vivo [29], however, this application is still in its infancy and future studies will reveal whether ROS-targeted PET is useful in PD patients. Advanced molecular imaging applications have led to considerable progress in our understanding of non-dopaminergic receptor systems, including glutamatergic, cholinergic, serotonergic and adrenergic signaling in PD [10, 30, 31].

PD can be successfully managed for many years following disease onset. Nonetheless, while the oral administration of L-dopa is still considered the cornerstone of PD therapy, chronic L-dopa treatment is associated with several limitations, including drug-induced dyskinesia, motor fluctuations, and hallucinations [32, 33]. Further, PD remains a progressive disease with no curative treatment to date, which ultimately leads to severe disability. Accordingly, the development of improved PD therapy constitutes an unmet medical need and various therapy concepts have been suggested in addition to dopamine substitution. Contemporary efforts aim at introducing novel disease-modifying drugs that harbor potential to delay disease progression.

Innovative concepts involving translational molecular imaging have proven particularly useful for the early detection of PD and can facilitate the identification of pre-symptomatic individuals at risk. Similarly, strenuous efforts have been devoted to the development of molecular probes that would facilitate drug discovery and development by enabling the non-invasive assessment of molecular processes, which may include alterations in receptor expression for target validation and therapy monitoring, drug biodistribution and pharmacokinetics, target engagement experiments, and micro-dosing studies [34–36].

Molecular imaging tools have proven valuable, from bench-to-bedside, and may effectively reduce late-stage failures in drug development. Imaging modalities such as positron emission tomography (PET) and single photon-emission tomography (SPECT) can provide real-time information on drug distribution in patients as well as the assessment of target protein abundancy in different organs without physiological disturbance of the tissue microenvironment [37]. Indeed, PET and SPECT have been applied to study PD and were established as reliable clinical tools that provide information on PD onset and progression (Fig. 1C) [38, 39]. In most cases, targeted PET probes can be used for multiple purposes, including pharmacological assessment by means of target engagement studies, as well as for diagnostic purposes when alterations in target abundancy are anticipated. Another key advantage of molecular imaging-guided drug development is the ability to provide effective patient stratification during clinical PD trials [40–43].

In this review, we discuss key concepts that led to the discovery of translational molecular imaging probes and therapeutic agents for PD. Further, this review sheds light on current developments, thereby discussing potential novel diagnostic tracers and experimental disease-modifying drug candidates.

## Targeting α-synuclein

As PD progresses, protein pathology spreads from the brainstem to the substantia nigra pars compacta (SNpc) and then to the forebrain [13]. Although a variety of molecular mechanisms are believed to contribute to PD onset and its irreversible progression, the most extensively studied pathological hallmark of PD is the accumulation of intracellular inclusion bodies (Lewy bodies), that consist of misfolded α-synuclein (αS). Of note, αS is a 140 amino acid long native presynaptic neuronal protein that is encoded by the *SNCA* gene [44] and is abundantly expressed in the healthy CNS, where it regulates vesicle fusion and neurotransmitter release via molecular interactions with presynaptic soluble *N-*ethylmaleimide-sensitive attachment protein receptors (SNAREs) [45]. Misfolding of αS leads to the formation of aggregates of the protein, and such aggregates are implicated in a group of overlapping neurodegenerative disorders, designated α-synucleinopathies, which include PD, dementia with Lewy bodies (DLB) and multiple system atrophy (MSA) [46–49]. Genetic mutations, post-translational modifications (PTMs), and increased αS protein concentrations have been linked to the formation of insoluble αS aggregates [50]. One of the earliest PD-related mutations discovered were within the *SNCA* gene, which was found to initiate early-onset PD with an autosomal dominant inheritance pattern [51]. Some advances have been made since then by the identification of a series of mutations in the αS gene that were linked to hereditary PD. *A30P, E46K, H50Q, G51D, A53E, A53V* and *A53T* are mutations in the αS gene that were linked to hereditary PD [52–60]. Similarly, several PTMs, such as phosphorylation, ubiquitination and nitration, have emerged as valuable markers of αS pathology [61–63]. Even though today's medications against PD provide symptomatic relief at early disease stages, there is a need to develop drugs that instead target the underlying disease process and thereby can delay or halt disease progression.

**Fig. 2:**
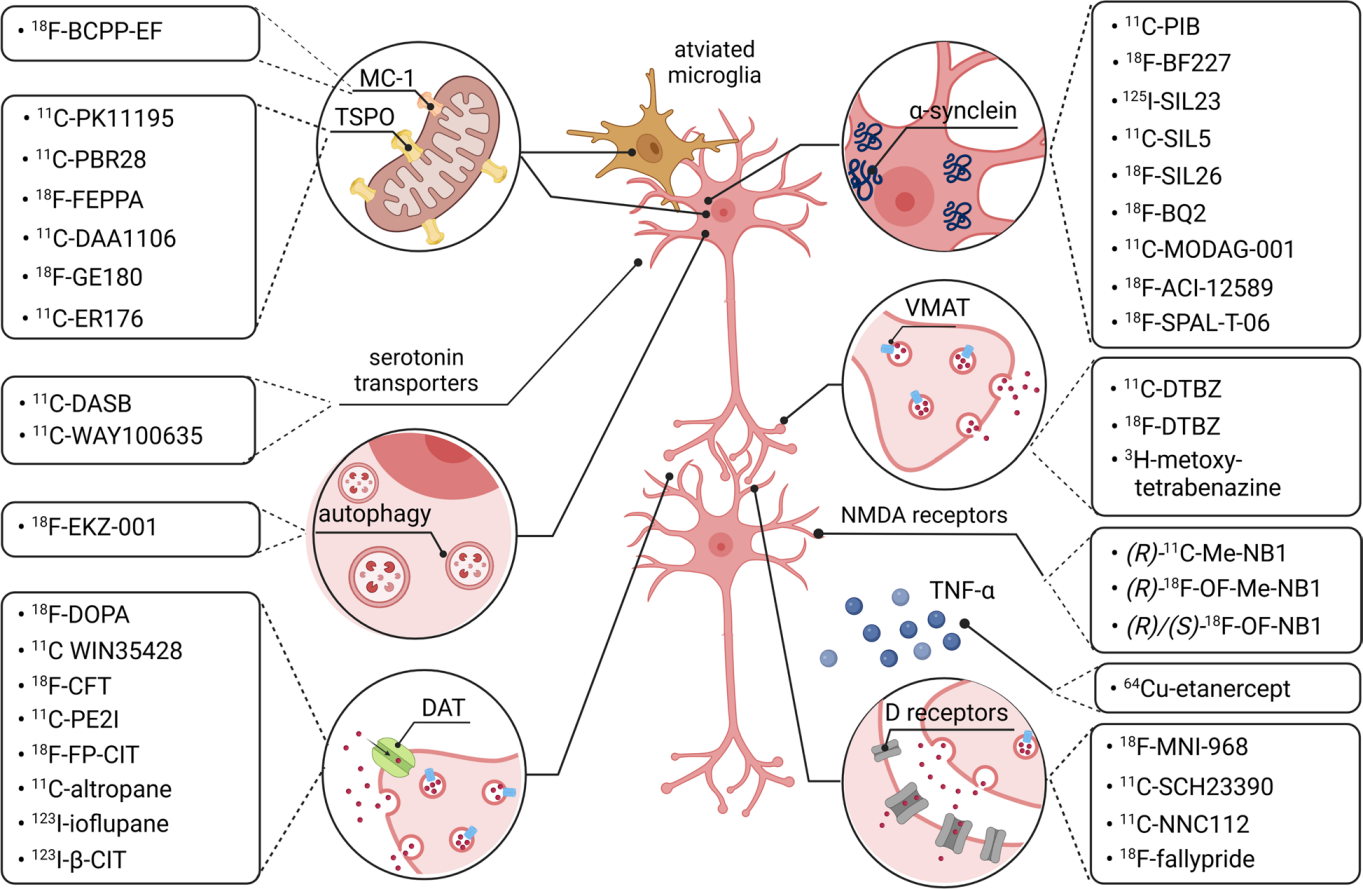
Molecular imaging in Parkinson’s disease (PD). Selected biological targets that have been harnessed for positron emission tomography (PET) and single photon-emission tomography (SPECT) imaging in PD. These targets have been used to allow diagnostic imaging as well as to facilitate drug discovery & development and include the translocator protein 18 kDa (TSPO), which is upregulated in activated microglia, mitochondrial complex I (MC-I) to assess mitochondrial function, various α-synuclein and amyloid-β (Aβ)-targeted probes, radiolabeled dopamine & serotonin transporter substrates & ligands of dopamine receptors, probes targeting the vesicular monoamine transporter 2 (VMAT2) and attempts to visualize autophagy as well as cytokines such as the tumor necrosis factor α (TNF-α). Given that these molecular variables are critical to the pathophysiology of PD, most of them have been suggested as therapeutic targets

There is much interest in non-invasive molecular imaging of αS, with the hope that αS detection could serve as a biomarker for presymptomatic detection of PD, or for monitoring of disease progression. Although the development of αS-targeted probes is still in its infancy, there have been attempts to develop high-affinity ligands with appropriate tracer pharmacokinetics. An overview of selected αS-targeted translational molecular imaging probes is provided in Fig. 2. Initially, probes that were known for their use in Alzheimer’s disease were tested in PD patients. Indeed, amyloid-beta (Aβ)-targeted probes, such as the thioflavin-T derivative, ^11^C-Pittsburgh compound-B (^11^C-PIB), and benzoxazole BF227 (^18^F-BF227), have been suggested to also be useful for the early diagnosis of PD since they not only bind to Aβ, but also to the beta-sheet structures of αS-aggregates [64]. However, recent studies with brain homogenates revealed that ^11^C-PIB binding was not associated with the presence of Lewy bodies [65, 66]. Similarly, the radiofluorinated amyloid PET ligand, ^18^F-BF227, did not show meaningful binding to brain homogenates enriched with Lewy bodies [67, 68]. These findings encouraged the development of novel αS-selective PET tracers beyond repurposed Aβ-targeted probes. Along this line, Yu et al. synthesized a series of phenothiazine derivatives and reported the discovery of SIL5, SIL23, and SIL26 as high-affinity ligands for αS fibrils [69]. The same group subsequently reported the synthesis of the ^125^I-labeled analogue of SIL23, ^125^I-SIL23, which exhibited specific binding to αS fibrils in postmortem PD brain tissue as well as on brain slices of αS transgenic mice [70]. Despite these encouraging *in vitro* findings, the further development of ^125^I-SIL23 was plagued by a high degree of non-specific binding *in vivo* [70]. Structural derivatization of ^125^I-SIL23 yielded ^11^C-SIL5 and ^18^F-SIL26 with improved selectivity, however, their pharmacokinetic properties suggested the need for further optimization to achieve successful αS imaging *in vivo* [71]. The development of novel PET imaging agents for PD has been increasingly focusing on the discovery of high-affinity αS-targeted probes with suitable *in vivo* kinetics and biodistribution, which led to the design of novel scaffolds via high throughput screening [72, 73]. Among these scaffolds, *N, N-*dibenzyl-cinnamamide [74] and bisquinoline derivatives such as ^18^F-BQ2 [75] and ^11^C-MODAG-001 [76] proved to be promising lead candidates with moderate-to-high brain uptake, laying the foundation for the development of suitable clinical candidates. Matsuoka et al., recently reported on the clinical translation of an αS-targeted PET ligand codenamed ^18^F-SPAL-T-06, which was co-localized with regions known to accumulate glial cytoplasmic inclusion bodies in multiple system atrophy (MSA) patients [77]. Similarly, the biotech company AC Immune has recently announced that their lead αS-targeted PET ligand, ^18^F-ACI-12589, revealed excellent *in vivo* performance characteristics, supporting its translation into humans [78]. Future studies will examine the suitability of ^18^F-ACI-12589 to image αS aggregates in patients with PD or  MSA.

Targeting αS with radiolabeled antibodies constitutes a challenge due to the low penetration of antibodies across the blood–brain barrier. To overcome this limitation, more recent examples of αS-targeted PET imaging involve the use of bispecific antibodies that carry a transferrin binding domain, to support blood–brain barrier penetration via receptor-mediated transcytosis, as well as a high-affinity αS binding domain [79]. Probing αS is further complicated by posttranslational modifications, which may affect protein folding and capacity to induce neuronal damage. Indeed, it was found that the serine residue at position 129 (Ser 129) of αS is selectively and extensively phosphorylated in synucleinopathy lesions. Phosphorylation of αS at Ser 129 promoted fibril formation in vitro, pointing towards a key role of this particular modification in the pathogenesis of α- synuclopathies [80], and prompting calls to carefully consider the impact of posttranslational modifications on the pharmacodynamic properties of PET and SPECT imaging probes. It should be noted that dementia is increasingly recognized as a common consequence of PD and around 50% of PD dementia cases develop significant amounts of Aβ plaques or p-tau containing neurofibrillary tangles [81]. As such, the specificity and selectivity of αS-targeted probes over these other proteinopathies is of paramount importance to decrease the likelihood for false positive scans, which constitutes an ongoing challenge for contemporary probe development. Other challenges in tracer development include the notion that αS is not abundantly expressed at early PD stages, requiring a highly sensitive detection tool and hampering the development of a suitable αS-targeted imaging probe for early PD diagnosis. Notably, αS aggregate structure, as well as spatial brain distribution and abundance of αS can vary depending on the underlying α-synucleinopathy, adding more layers of complexity, but concurrently providing an opportunity to harness these differences for differential diagnosis. A recent study concluded that the intrinsic structure of αS fibrils determines the characteristics of synucleinopathies [82]. For instance, fibrillar αS assemblies derived from patients with MSA exhibited strong similarities with PD-derived αS fibrils, but were significantly more potent in prompting nigrostriatal neurodegeneration, spread of αS pathology, inflammation and motor deficits, reflecting the aggressive nature of this disease [82]. In sharp contrast, strains from DLB cases displayed very modest neuropathological features. DLB is clinically distinguished from PD dementia by the timing of dementia onset – with individuals that develop dementia up to one year after the onset of motor symptoms considered to suffer from DLB, and individuals developing dementia later than one year following the onset of motor symptoms being assigned to the PD dementia group [83, 84].

Despite these various challenges in the development of αS-targeted PET ligands, a clinically validated probe could be used as a biomarker for disease progression, as well as to monitor therapy response in future trials. Further, other approaches such as real-time quaking-induced conversion (RT-QuIC) have enabled the identification of misfolded αS in the cerebrospinal fluid (CSF) of patients with prodromal and manifest α-synuclopathies with a sensitivity of 90–95% and a specificity of 80–100% [85, 86]. While the latter diagnostic performance is intriguing, limitations of current RT-QuIC applications for the detection of misfolded αS include the invasive nature of lumbar puncturing as well as the lack of information on spatial distribution of Lewy bodies across different brain regions. Finally, given the involvement of peripheral organs in PD, more recent findings indicated that colon, skin, gastrointestinal and submandibular gland biopsies may constitute valid alternatives to the brain as a source of pathological αS for diagnostic purposes [87–92]. A recent study by Wang et al. concluded that αS seeding activity from skin samples can serve as a novel biomarker for antemortem diagnoses of synucleinopathies [93]. Employing RT-QuIC analysis to assess 41 antemortem skin biopsies from patients with PD and controls, the authors found that posterior cervical and leg skin biopsy tissues provided a diagnostic sensitivity and specificity for the detection synucleinopathies in PD patients of 95% (95% CI, 77–100) and 100% (95% CI, 84–100), respectively. Along this line, phosphorylated αS depositions within autonomic nerve terminals of biopsied skin samples have been detected by immunohistochemistry (IHC) and immunofluorescence (IF) microscopy in living PD patients. Although a variety of immunohistochemical assays have been reported, the majority of studies were conducted at single centers without blinded and independent replication. However, in a recent study by Donadio et al., skin biopsies have been validated across laboratories, showing a high degree of reproducibility [94]. In most reported cases, peripheral biopsies were successfully used to discriminate PD patients from healthy subjects. Larger multicenter trials are warranted to unleash the full diagnostic potential of peripheral PD biopsies. Further, molecular imaging probes with tailored pharmacokinetic properties may facilitate the non-invasive assessment of pathological αS in peripheral organs such as the colon or submandibular gland.

The availability of molecular imaging tools to visualize αS has the potential to support emerging therapeutic concepts that are focused on attenuating the formation of misfolded αS aggregates, through reduction of αS production, inhibition of αS aggregation, increased αS degradation, or inhibition of uptake of extracellular αS into neurons [95]. For instance, reduced αS expression can be achieved by the silencing of the *SNCA* gene via small interfering RNA (siRNA). Studies have established the potential neuroprotective effects of siRNAs in rodent models of α-synucleinopathies, particularly upon intracranial administration in hippocampal and cortical regions of the rodent brain [96–98]. In a recent study by Mittal et al., β2-adrenoreceptors were found to regulate *SNCA* transcription via histone 3 lysine 27 acetylation of its promoter and enhancers [99]. Along this line, individuals treated with β2-adrenoreceptor agonists had a significantly reduced risk of developing PD (OR = 0.66, 95% CI, 0.58 to 0.76) [99]. Further, studies have highlighted the potential of heat shock proteins (HSPs) [100, 101]. Of note, HSPs serve as molecular chaperons, capable of preventing the aggregation of misfolded proteins such as pathological αS. It was found that the small heat-shock proteins, αB-crystallin and Hsp27, prevent αS aggregation in vitro via transient molecular interactions that were dependent on the kinetics of αS aggregation [101]. Similarly, Hsp70 was found to reduce the amount of aggregated αS in vitro and in vivo, in an α-synucleopathy mouse model overexpressing Hsp70, protecting it from αS-dependent neurotoxicity. Further, first-generation epitope vaccines that were generated against B cell antigenic determinants of human αS and produced high titers of anti-human αS antibodies attenuated Lewy bodies pathology in brain specimens from DLB cases [102]. Active and passive immunization against α-syn have shown neuroprotective activity via attenuation of αS aggregation in transgenic mouse models of PD [103–105]. In an initial clinical trial, the humanized IgG1 monoclonal antibody, prasinezumab, prompted a 96.5% reduction of soluble serum αS with good tolerability and favorable safety profile in patients [106]. However, results of a phase 2 clinical trial (NCT03100149) showed that the drug has limited effects on disease progression [107]. To date, interesting exploratory concepts of αS-targeted therapy involve the development of monoclonal antibodies against αS, which promote its degradation [105]. Further, the anti-αS antibody, Lu-AF82422, has proven to inhibit seeding of αS in preclinical studies [108] and may constitute a promising candidate for the treatment of α-synucleinopathies in future clinical trials. Of note, drug development efforts in Alzheimer’s disease revealed that immunotherapies directed at aggregated forms of Aβ, with the particular aim not to target monomeric Aβ, proved to be highly promising [109, 110]. Similar attempts to design and develop antibodies that specifically target aggregated oligomeric forms of αS are currently emerging [111–113], and future studies will reveal their therapeutic potential. Given their neurotoxic properties, oligomeric αS forms are considered a particularly attractive target for passive immunization strategies in PD [114]. As such, several examples have been reported in which antibodies against oligomeric αS triggered a reduction in αS accumulation in studies with transgenic murine models of synucleinopathies, as recently reviewed by Vaikath et al. [115]. Similar to the examples from PET ligand development, bispecific antibodies that undergo transferrin receptor-mediated transcytosis have also been developed for therapeutic purposes [116]. This concept has been leveraged to advance Aβ-targeted antibodies to AD patients and has found application in preclinical PD studies, as evidenced by the reduction of αS pathology in transgenic L61 mice following treatment with the bispecific antibody, RmAbSynO2-scFv8D3 [117]. It is anticipated that similar efforts will be used in clinical trials with PD patients in the near future. Overall, αS-targeted therapy in PD has been extensively investigated in preclinical models and a steadily accumulating body of clinical evidence seems to underscore the potential value of development of αS-targeted therapy.

## Mitochondrial and lysosomal dysfunction

The link between parkinsonism and mitochondrial dysfunction was initially established when the neurotoxin, *N-*methyl-4-phenyl-1,2,3,6-tetrahydropyridine (MPTP), was reported to trigger acute parkinsonism in humans [118], with similar observations in rodents and non-human primates [119–122]. These observations gave rise to concerted research into the role of mitochondrial dysfunction in PD. Of note, MPTP is a pro-drug that rapidly passes through the blood–brain barrier. Once in the brain, it is oxidized to the active compound, 1-methyl-4-pyridinium (MPP +). MPP + is a substrate of the dopamine transporter, thus leading to MPP + accumulation in dopaminergic cells, where the compound inhibits respiration at mitochondrial complex I (MC-I) of the electron transport chain [123–125]. Abnormal mitochondrial dynamics, including fission and fusion, was found to prompt dopaminergic nerve loss, thus providing a potential mechanism of MPTP-induced parkinsonism [126]. Later studies showed that many PD-associated gene mutations directly or indirectly lead to mitochondrial dysfunction, [127–129] which leads to the formation of reactive oxygen species (ROS), exposing the respective neurons to persistent oxidative stress [47, 57, 58]. Although MC-I deficiencies are the primary source of ROS in PD [125], other factors including auto-oxidation of dopamine [130] and neuroinflammation also contribute to oxidative stress [21]. Given (1) the implications of mitochondrial dysfunction and oxidative stress in PD and (2) the critical role of MC-I in maintaining redox homeostasis, targeting MC-I may constitute a promising diagnostic and therapeutic approach in PD. Indeed, MC-I selective PET ligands such as ^18^F-BCPP-EF and ^18^F-flurpiridaz have been developed and validated in humans [131, 132]. These tools can be readily harnessed to facilitate the discovery of MC-I-targeted therapeutic agents, thereby harboring the potential to provide a novel disease-modifying concept in PD. It should be mentioned, however, that ^18^F-flurpiridaz is mainly used for myocardial perfusion imaging due to the perfusion-dependent tracer kinetics in the myocardium [133, 134]. It is currently unclear whether ^18^F-flurpiridaz can be leveraged for mapping MC-1 in the mammalian brain. Beyond the role of MC-I in mitochondrial dysfunction, mutations in *PINK1*, the gene encoding for PTEN-induced putative kinase 1 (PINK1), are associated with mitochondrial dysfunction in PD, whereas patients who are heterozygous for mutations in *PINK1* present with reduced intensity on ^18^F-fluoro-levodopa PET compared with controls [135].

Lysosomal dysfunction has been increasingly recognized as another key determinant in the pathophysiology of PD. Along this line, the lysosomal hydroxylase glucocerebrosidase (GCase), encoded by the GBA gene, is known to exhibit pathogenic mutations that can cause hereditary PD [136]. GCase and αS form a bidirectional pathologic loop where GCase deficiency leads to a decline of lysosomal proteolysis that affects αS, while increased levels of αS attenuate the function of GCase in neuronal lysosomes [137, 138]. Targeting GCase as a therapeutic strategy for PD has been suggested. Ambroxol is considered a potentially disease-modifying agent that proved to increase brain GCase activity and decrease αS in experimental parkinsonism, prompting its clinical translation. Indeed, ambroxol has recently been advanced to clinical trials for the treatment of PD (NCT02941822) and PD-associated dementia (NCT02914366) [136, 139].

Similar to GCase, leucine-rich repeat kinase 2 (LRRK2) is involved in hereditary PD [140]. Genetic and biochemical data unveiled that hyperactivity of LRRK2 is linked to PD progression and thus it has been hypothesized that lowering its activity may exhibit disease-modifying effects in PD [141]. While attempts to develop LRRK2-targeted molecular imaging probes have been reported, a clinically validated PET or SPECT tracer is currently lacking [142–144]. Nonetheless, the recently reported radiofluorinated probe, ^18^F-PF-06455943, exhibited favorable performance characteristics in rodents and non-human primates, rendering it a promising candidate for clinical translation [145]. The availability of such a tool would facilitate the development of LRRK2-targeted therapeutics in the pipeline. Although LRRK2 kinase inhibitors have proven protective in experimental parkinsonism and have been suggested for clinical translation [146], the mechanisms by which mutant LRRK2 contributes to PD pathophysiology are not fully understood. LRRK2 does not form aggregates in the affected PD brain, however, the pathological function of mutated LRRK2 may involve common pathways with αS. For instance, mice that were transgenic for mutant human LRRK2 and overexpressed mutant human *SNCA* exhibited an enhanced deposition of α-synuclein, as compared with control mice that overexpressed mutant human *SNCA* alone [147]. In contrast, removal of the endogenous *Lrrk2* gene limited the detrimental effects of mutant α-synuclein, reducing protein deposition and neuronal loss in experimental parkinsonism [148]. Studies assessing PD-related changes in LRRK2 levels are limited. Nonetheless, a study that used laser microdissection to isolate dopaminergic neurons from the substantia nigra pars compacta of subjects with idiopathic PD and controls suggested that LRRK2 mRNA levels were attenuated in PD patients [149]. These results warrant confirmation in larger trials and may support the notion that LRRK2 could serve as a disease biomarker that can be targeted with molecular imaging probes. While the molecular mechanism underlying a beneficial effect of LRRK2 inhibitors in PD are not entirely understood, an LRRK2-induced attenuation of autophagy has recently been proposed [150]. Among the LRRK2 inhibitors in clinical development, DNL201 and DNL151 were well tolerated in a phase I study in healthy volunteers [146]. Further, DNL201 was evaluated in a phase Ib trial assessing drug safety and pharmacokinetics in 28 patients with early PD. DNL201 was generally well tolerated and demonstrated a robust cerebrospinal fluid penetration [151]. Other LRRK2 inhibitors such as MLi-2 and PF-06685360 have shown encouraging preclinical efficacy, however, there are no reports on human use of these agents to date [152]. Another approach to suppress LRRK2 activity is through the use of anti-sense oligonucleotides (ASOs) that can decrease LRRK2 expression. Indeed, the recently reported ASO, BIIB094, prompted a decrease of αS inclusions in mice, and its safety, tolerability and pharmacokinetic profile are currently being evaluated in humans (NCT03976349) [153, 154].

While PD has been linked to dysfunctional autophagy, attempts to target autophagy by translational molecular imaging probes have been scarce. The histone deacetylase 6 (HDAC6) constitutes a potential biological target to non-invasively assess autophagy. Indeed, HDAC6 was found to orchestrate autophagosome maturation [155] and prevent neurodegeneration by enhancing autophagy when the ubiquitin–proteasome system is impaired [156]. HDAC6 was further found to participate in PD pathophysiology [157], and can be targeted using ^18^F-EKZ-001 PET, as recently shown in a first-in-human study [158]. Indeed, ^18^F-EKZ-001 was well tolerated and exhibited favorable tracer pharmacokinetics, including rapid brain uptake and retention in cortical regions with high HDAC6 abundancy [158]. This imaging tool can be harnessed for clinical target engagement studies, facilitating the development of HDAC6 modulators in the pipeline. Moreover, future studies will reveal whether HDAC6-based molecular imaging is useful to assess the integrity of the autophagy machinery in PD.

## Neuroinflammation in Parkinson’s disease

**Fig. 3:**
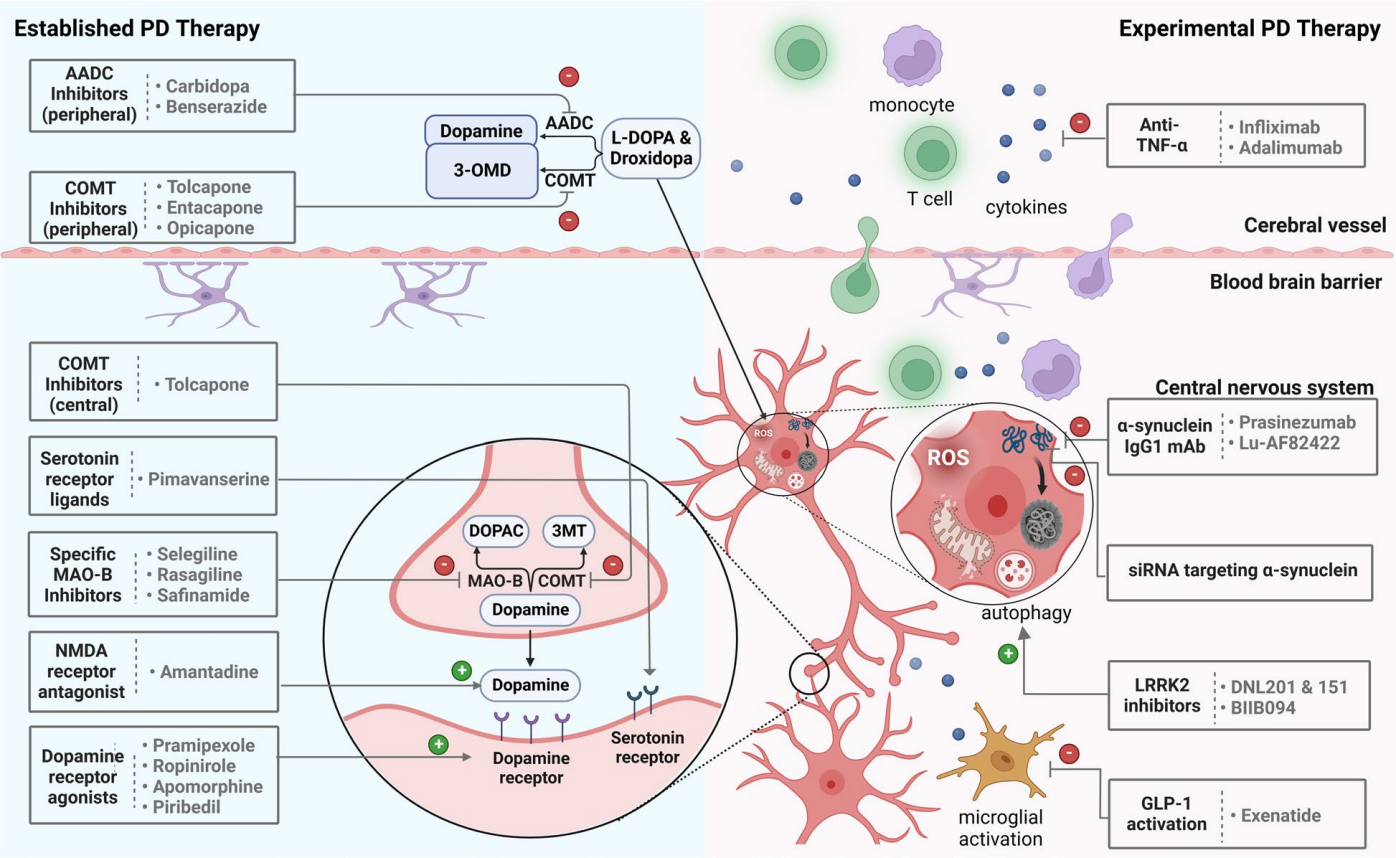
Established vs. experimental therapies in Parkinson’s disease (PD). While current drug PD therapy is primarily symptomatic and attempts to restore dopaminergic function, many lines of experimental research focus currently on the development of potentially disease-modifying concepts. The latter involve various targets that can be visualized using translational molecular imaging probes, thus providing the opportunity to conduct imaging-guided preclinical and clinical studies in PD. Among the promising concepts, preventing the formation of inclusion bodies by targeting misfolded α-synuclein, attenuating neuroinflammation via anti-tumor necrosis factor α (TNF-α) antibodies and glucagon-like peptide 1 (GLP-1) activation, as well as stimulation of autophagy with leucine-rich repeat kinase 2 (LRRK2) inhibitors have been suggested

Neuroinflammatory mechanisms that contribute to the neuronal degeneration in PD include, but are not limited to, microglial activation [159], excessive cytokine release, and lymphocytic CNS infiltration (Fig. 3) [160]. The latter triggers deleterious events that ultimately result in cytokine receptor-mediated apoptosis, which, in turn, may contribute to the dopaminergic cell death and PD progression [22]. Microglia represent 10–20% of all glial cells in the brain and act as a natural defense mechanism against pathogens and as key mediators of immune response in the brain [161, 162]. However, excessive microglial activation following mitochondrial dysfunction and oxidative stress can ultimately result in neuronal cell death. Upon activation, microglial cells upregulate the peripheral benzodiazepine receptor (translocator protein 18 kDa, TSPO) – an essential component of the mitochondrial membrane that mediates functions such as cholesterol transport, mitochondrial respiration, apoptosis, and cell proliferation [163]. Although the underlying trigger of increased TSPO expression in activated microglia is not fully understood, TSPO is believed to be a key modulator of microglial activity [164]. TSPO has been used as a biomarker for neuroinflammation and a variety of clinically validated imaging probes for TSPO have been developed (Fig. 2). The isoquinoline derivative, ^11^C-PK11195, was the first TSPO PET ligand that proved to be clinically useful for imaging neuroinflammation [165, 166]. PET studies indicated that there is pronounced microglial activation in various regions of the PD brain, as evidenced by studies with ^11^C-PK11195 [167, 168]. However, due to the limitations of ^11^C-PK11195, which include high non-specific binding, low brain penetration, and high plasma protein binding, the clinical use of ^11^C-PK11195 is hampered [169, 170]. Second generation radiotracers with superior imaging characteristics have subsequently been developed, including several probes with variable chemical structures, such as ^11^C-PBR28, ^18^F-FEPPA, ^11^C-DAA1106 [171–179]. One critical drawback of these tracers is their sensitivity to polymorphism in the TSPO gene, thus affecting their binding properties and leading to increased inter-individual variability. The latter shortcoming prompted the development of third generation TSPO-targeted probes such as the tricyclic indole, ^18^F-GE180 [180]. The ability of ^18^F-GE180 to bind selectively and with high affinity to TSPO is critical for the assessment of neuroinflammation in disease states, providing significantly better TSPO imaging signal to noise ratios and lower nonspecific binding, as compared to ^11^C-PK11195 [181, 182]. Notably, it was recently suggested that substantial discrepancies between in vitro and in vivo sensitivity to TSPO gene polymorphism might further complicate TSPO PET ligand development, as evidenced by studies involving the translational work on quinazoline-2-carboxamide derivative, ^11^C-ER176 [183], of which a radiofluorinated analogue (^18^F-**3b**) has been reported. [184] Future clinical trials are warranted to assess the utility of ^18^F-**3b** and ^18^F-GE180 to visualize neuroinflammatory processes in PD.

Other mechanisms by which microglial function can be attenuated include the activation of glucagon-like peptide-1 (GLP-1) receptor and the inhibition of inflammatory cytokines such as the tumor necrosis factor α (TNFα) [185, 186]. In a recent single-center, randomized, double-blind and placebo-controlled trial, patients with moderate idiopathic PD received the GLP-1 receptor agonist, exenatide, once weekly for 48 weeks in addition to their regular medication, prompting a significant improvement of their clinical scores, as compared to the placebo group [185]. These results not only suggested that exenatide may warrant further study as a therapy, but also provided the opportunity to pursue imaging-guided clinical trials with radiolabeled exenatide analogs, which have recently entered the clinical arena [187–189]. While anti-TNFα antibody therapy with agents such as with infliximab or adalimumab has been established for peripheral inflammatory diseases [190], these agents have not yet been extensively studied as potential therapeutics in PD. In the wake of previous examples of bispecific anti-TNFα antibodies, it is conceivable that a bispecific anti-TNFα antibody, that allows receptor-mediated transcytosis, may be of particular interest for therapy studies in PD patients. Further, the notion that ^64^Cu-etanercept was shown to rapidly accumulate in the choroid plexus and the CSF within the cerebral ventricles of a living rat after peripheral administration [191] points towards a potential utility of the probe to visualize TNFα in the central nervous system. The latter may lay the foundation for the assessment of TNFα abundancy in live PD patients in the near future, as well as for the potential assessment of target engagement by anti-TNFα drugs in the PD brain.

Triggering receptor expressed on myeloid cells 2 (TREM2) is heavily implicated in glial function and axonal myelination [192]. Genetic variants of TREM2 were linked to PD, however, the potential role of TREM2 on the formation and aggregation of pathological αS is poorly understood. In a recent study involving BV2 microglial cells and TREM2 knock-out mice, it was demonstrated that TREM2 deficiency aggravates αS-related neuropathology [193]. Further, soluble TREM2 levels were found to be elevated in PD subgroups presenting with increased levels of tau in the CSF [194]. Further studies to identify molecular pathways linking TREM2 with αS are required to pave the way for TREM2-targeted novel therapeutic concepts in PD. Similarly, molecular imaging probes directed at TREM2 [195] hold promise to serve as research tools, thereby shedding light on the role of TREM2 in PD pathophysiology.

## Dysregulation of neuroreceptor systems

To date, most of the routinely used antiparkinsonian medications target dopamine deficiency. While dopaminergic pharmacological treatment is presumed not to considerably affect disease progression, it has enabled sustained symptom control and ground-breaking improvements in the quality of life for PD patients [13]. An overview of the contemporary arsenal of validated and selected experimental PD drugs is provided in Fig. 3.

While progressive degeneration of dopaminergic neurons is evident in PD, experimental and clinical studies have highlighted the involvement of other neuroreceptor systems, including the glutamatergic, serotonergic, adrenergic and cholinergic system [10, 196]. For instance, it has been suggested that a well-defined balance between central dopaminergic and cholinergic signaling is required for movement control and that efforts to restore that balance, with anticholinergic agents, would result in improvement of motor symptoms in PD patients [197, 198]. Approved anticholinergics provide symptomatic relief for tremor and to some extent for rigidity, however, they exhibit significant side effects (such as memory disturbances or somnolence), which makes them unusable in most elderly parkinsonian patients. Although dopaminergic therapy has largely supplanted the use of anticholinergic drugs, they are still used in clinical practice, particularly in younger PD patients with prominent tremor [199–202].

Aside from the CNS implications of the cholinergic system in PD, there is a solid body of evidence supporting its role in autonomic dysfunction in peripheral organs, which is observed in most PD patients and substantially affects their quality of life. Peripheral PD symptoms (cholinergic or non-cholinergic) include gastrointestinal dysfunction, cardiovascular dysregulation, urinary disturbances, sexual dysfunction, and thermoregulatory changes [203, 204]. Intriguingly, αS pathology has recently been linked to autonomic denervation [205–208]. Cardiac autonomic denervation has the potential to allow differential diagnosis between PD and other forms of parkinsonism, thus rendering it a useful biomarker for diagnostic imaging [209]. A summary of contemporary molecular imaging concepts involving dysregulated neuroreceptor systems in PD is provided in Fig. 2.

### Dopamine receptors

Postsynaptic dopamine receptors are divided into five subtypes, namely, dopamine receptors 1–5 (D1-D5). While D1 and D5 are coupled to G stimulatory sites and activate adenylyl cyclase, D2-D4 couple to G inhibitory sites, thereby inhibiting adenylyl cyclase [210]. Dopamine receptors are typically upregulated in early PD, which can be quantified by dopamine receptor PET [211, 212]. A major obstacle of D1-targeted PET imaging has been the challenging development of selective probes. While ^11^C-SCH23390 was initially identified as D1 PET ligand and was advanced to humans [213], the broad use of this tracer was hampered due to the limited subtype-selectivity. A close derivative, ^11^C-SCH39166 exhibited improved selectivity, however, at the expense of reduced binding affinity towards D1 [214]. Further, another PET ligand code-named, ^11^C-NNC112, was suggested for quantitative assessment of dopamine receptors, [215] but was demonstrated to have off-target binding at 5-HT2A receptors in humans [216]. Tamagnan et al. reported on the development of a potentially promising D1-targeted probe codenamed ^18^F-MNI-968, which was administered to non-human primates and humans [217]. Although preliminary data was deemed promising, further studies are currently ongoing to elucidate the potential of ^18^F-MNI-968 for subtype-selective imaging of D1 receptors. With regard to D2 receptor imaging, ^11^C-raclopride has been widely used. This agent competes with endogenous dopamine, thereby proving to be a useful tool for qualitative assessment of D2 receptor density [213]. Alternative D2-targeted PET probes such as ^18^F-fallypride are considered valid alternatives with improved binding affinities at D2 receptors [218].

These dopamine receptor-targeted probes are not currently in routine use for the diagnosis of PD because of potential interactions of dopamine replacement therapy with tracer-receptor binding, and the complexity of such analyses related to the dopamine receptor autoregulation in response to the loss of dopaminergic neurons. However, the imaging tools are valuable to facilitate the development of improved dopamine receptor agonists. Indeed, these tools have been harnessed to assess target occupancy by endogenous dopamine, or dopamine released in response to levodopa therapy, and various synthetic agonists across different species, thereby advancing our understanding of the biologic effects of dopaminergic therapy [219–223].

Various dopamine receptor agonists have been developed and shown to attenuate PD motor symptoms. Dopamine receptor agonists can be divided in two major classes, namely, ergot- and non-ergot derivatives. Because the use of ergot derivatives has been associated with pleuropulmonary and cardiac valvular fibrosis [13], currently used dopamine receptor agonists belong to the non-ergot family [224]. Examples include pramipexole, ropinirole, rotigotine, apomorphine and piribedil [225]. Although, they were initially licensed for synergistic administration with levodopa at advanced PD stages, their application as monotherapy has also proven useful in PD [224, 226]. In conclusion, targeting dopamine receptors constitutes a compelling example of how translational molecular imaging probes can be exploited to provide imaging-supported PD therapy.

### DOPA decarboxylase and dopamine transporters

In the mammalian brain, DOPA decarboxylase (DDC) facilitates the transformation of levodopa to dopamine [227]. Levodopa is the L-isomer of the naturally occurring amino acid, D,L-dihydroxy-phenylalanine [228]. Unlike dopamine, levodopa crosses the blood–brain barrier, thus allowing peripheral administration of the drug. The paramount therapeutic value of levodopa in PD was recognized when the enzyme that converts levodopa to dopamine, DDC, was found to be present in high amounts in the mammalian brain [229, 230]. For more than half a century, levodopa has been the first-line treatment for PD [231–233]. Indeed, levodopa therapy is remarkably effective in attenuating primary motor symptoms of PD patients.

Upon oral administration of levodopa, it undergoes an extensive first-pass metabolism and rapid plasma clearance, allowing for only 1% of the administered dose to enter the brain [234]. This limitation can be overcome by co-administration of peripheral DDC inhibitors that do not enter the central nervous system (CNS). In fact, the latter was shown to substantially increase CNS availability of levodopa, thus reducing the substantial peripheral side effects of levodopa treatment, and improving its central efficacy [234–238]. Moreover, administration of levodopa via inhalation (CVT-301) [239] or continuous subcutaneous infusion [240–242] – mainly to circumvent the first-pass effect – have been suggested to maintain a steady prodrug supply to the brain. Contemporary levodopa treatment regimens include the co-administration of a peripheral DDC inhibitor, carbidopa or benserazide, which inhibits the peripheral decarboxylation of levodopa, thus reducing peripheral toxicity (nausea) and allowing greater CNS bioavailability of levodopa [13]. To further improve the availability of dopamine in the brain of parkinsonian patients, inhibitors of levodopa (and dopamine) metabolism via catechol-ortho-methyl-transferase (COMT) can be used. COMT inhibitors such as entacapone, tolcapone and opicapone have been introduced as an adjunct to levodopa-based combination therapies [243, 244]. As opposed to DDC and COMT inhibitors, which primarily lead to inhibition of peripheral levodopa metabolism, monoamine oxidase B (MAO-B) inhibitors prevent the degradation of dopamine in the CNS [245]. As such, synaptically released dopamine can be cleared by oxidation via MAO-B, and pharmacological modulation of MAO-B with rasagiline, selegiline or safinamide has been shown to increase synaptic dopamine concentrations, thus rendering them useful as an adjunct to levodopa therapy, and also as early-stage monotherapies in PD [246–249].

Chronic levodopa therapy can be associated with the development of troublesome motor complications, specifically levodopa-induced dyskinesias (LID) and motor response fluctuations, including sudden wearing-off effects [250, 251]. The underlying mechanisms for the development of dyskinesias with chronic levodopa replacement therapy are not yet fully understood, however, non-physiological pulsatile dopamine receptor stimulation in the striatum is considered to be an important factor [13, 252]. Based on this concept, drug delivery approaches that are less encumbered by pulsatile changes in brain dopamine levels, including sustained-release formulations and continuous levodopa release through implanted pumps or intestinal gels have been used effectively to prevent or ameliorate the development levodopa-induced dyskinesias [253, 254].

Translational molecular imaging offers an effective way to evaluate the biodistribution of levodopa when administered via different application routes, such as those mentioned above. Similar to levodopa, the radiofluorinated analog, 6-^18^F-fluoro-levodopa (^18^F-FDOPA), is trapped in the brain by DDC-mediated biotransformation, rendering ^18^F-FDOPA a suitable tool to study drug biodistribution, dopamine storage capacity, as well as DDC activity and the loss of dopaminergic neurons in living subjects [255–258]. Similar to levodopa, ^18^F-FDOPA readily crosses the blood brain barrier through neuronal amino acid transporters and is taken up by presynaptic terminals of dopaminergic neurons via the dopamine transporter (DAT), where it is metabolized to ^18^F-dopamine by DDC and stored in synaptic vesicles (Fig. 2). [257, 259] ^18^F-FDOPA PET has been used to assess the degree of nigrostriatal dopaminergic nerve loss in PD patients [260, 261]. In combination with a clinical history, ^18^F-FDOPA is considered a reliable diagnostic tool for the early diagnosis of PD. In a recent clinical trial encompassing 56 patients with suspected PD, ^18^F-FDOPA was found to be highly specific and sensitive for the diagnosis of PD, which led to approval of its use for this purpose by the US Food and Drug Administration (FDA) in 2019 [262].

Other PET tracers that are considered substrates of DAT are typically employed for the assessment of presynaptic dopaminergic neurons in the nigrostriatal system [38]. In line with histopathological evidence, it was found that the uptake of DAT-targeted probes in the striatum is substantially reduced in PD patients, as compared to controls. Selected examples of reported DAT PET radioligands include ^11^C-WIN 35,428 (^11^C-CFT) [263], ^18^F-CFT [264], ^11^C-PE2I [265] and ^11^C-altropane [266]. All of these PET probes are highly sensitive to striatal dopaminergic denervation in PD. Similarly, DAT-targeted SPECT radioligands have proven useful for PD diagnosis. Particularly, ^123^I-ioflupane (the ligand used commercially for DaTscans) and ^123^I-β-CIT have been broadly used to support the assessment of parkinsonism [267]. While carbon-11 and fluorine-18 labeled DAT-targeted PET ligands typically provide higher image resolution than iodine-123 labeled SPECT tracer, the latter exhibit a longer physical half-life, which facilitates the logistics and distribution to facilities without on-site tracer production. In conclusion, DAT imaging with either PET or SPECT is the most widely applied functional imaging tool for the diagnostic work-up and can be considered a valuable tool for PD diagnosis in clinical routine [268–270].

### Vesicular monoamine transporter 2

While vesicular monoamine transporter 1 (VMAT1) is mainly expressed in the periphery, the central nervous system is endowed with a high abundancy of vesicular monoamine transporter 2 (VMAT2) [271]. There are several PET tracers available to image the distribution of VMAT2. The potentially most prominent VMAT2-targeted probes belong to the class of tetrabenazines and include tritium-labeled methoxytetrabenazine for preclinical applications, as well as ^11^C-dihydrotetrabenazine (^11^C-DTBZ) for preclinical and clinical PET imaging. ^3^H-methoxytetrabenazine was successfully used to map VMAT2 density in rodents [272]. Similarly, ^11^C-DTBZ has been used in humans to monitor the integrity of striatal monoaminergic nerve terminals [273]. To improve the half-live of ^11^C-DTBZ, a radiofluorinated analog, ^18^F-DTBZ (^18^F-AV-133), was developed and tested in PD patients [274]. VMAT2 binding, as determined by ^18^F-DTBZ PET, correlated significantly with clinical disease severity and duration [274, 275]. Collectively, these results suggested that ^18^F-DTBZ PET is a powerful tool to measure dopaminergic degeneration and monitor disease progression in PD patients.

### Serotonergic and glutamatergic system

Although most reported PET and SPECT tracers for use in PD are geared towards evaluating the function or integrity of the dopaminergic system, several non-dopaminergic targets have recently been suggested as molecular biomarkers in PD. While dopaminergic markers allow for the diagnosis of PD at intermediate disease stages (Braak stages ≥ 3), it is possible that non-dopaminergic biomarkers could identify PD-related pathophysiological changes prior to the onset of clinical symptoms (Braak stages 1–2). The serotonergic system has been implicated in PD-associated depression, which typically develops early in the course of disease. Nonetheless, given the relatively high prevalence of depression in the general population, it remains to be seen whether serotonergic changes in PD-related depression can be distinguished from those in non-PD-related depression. Early observations included the low levels of 5-hydroxyindolacetic acid, a serotonin metabolite, in the cerebrospinal fluid, as well as the reduced cortical 5-HT1A receptors, in (symptomatic) PD patients suffering from depression [276]. Investigating the serotonergic system in PD patients was facilitated by the availability of serotonin transporter (SERT) PET ligands such as ^11^C-DASB [277] and ^11^C-WAY100635 [165, 278, 279]. Recently, serotonergic brain imaging with ^11^C-DASB has proven effective in identifying individuals at risk to develop PD, prior to the development of dopaminergic pathology and motor symptoms, which highlights the potential of imaging the serotonergic system for risk-stratification [280]. To date, pimavanserin, a serotonin 5-HT2A and 5-HT2C receptor ligand with antipsychotic effects, is approved by the FDA for the treatment of hallucinations and delusions associated with PD psychosis [281]. Further, conventional antidepressant therapy with selective serotonin reuptake inhibitors (SSRIs) and selective noradrenaline reuptake inhibitors (SNRI) are commonly used in PD patients suffering from depression.

Glutamate receptors (GluRs) are multimeric ligand-gated cation channels that mediate excitatory neurotransmission in the CNS. Given the role of glutamate receptors in modulating neurotransmission in the basal ganglia, it was suggested that drugs acting at these receptors may have antiparkinsonian effects [282]. GluRs are classified into ionotropic (iGluRs) and metabotropic glutamate receptors (mGluRs). IGluRs can be further subdivided into N-methyl-d-aspartate (NMDA), amino-3-hydroxy-5-methyl-4-isoxazolepropionic acid (AMPA) and kainate (KA) receptors, and the family of mGluRs constitutes 8 different subtypes, mGluR1-8 [279, 283]. Several NMDA antagonists, including competitive antagonists, noncompetitive antagonists, and glycine site antagonists were found to reverse muscle rigidity in rodent models of PD [282, 284]. Anti-parkinsonism effects of NMDA antagonists were also observed in other animal models of PD, including MPTP-treated non-human primates [285, 286]. Along this line, co-administration of NMDA antagonists with levodopa was found to potentiate the levodopa effect and decrease LID [282, 287]. Similarly, amantadine, a drug with non-competitive NMDA receptor antagonist properties, ameliorates LID [288]. Due to the possible side effects that arise from global inhibition of NMDA receptors, recent drug discovery efforts are focused on the identification of subtype-selective NMDA antagonists, for instance by targeting the GluN2B subunit of the NMDA receptor, which is believed to be a key contributor to excitotoxicity-induced neuronal cell death [289–291]. The recent breakthroughs in translational molecular imaging of GluN2B-containing NMDA receptors may help with the development of subtype-selective NMDA antagonists for the treatment of PD [292–300]. The most advanced class of GluN2B-targeted PET probes is based on a benzazepine scaffold and includes the carbon-11 derivative, *(R)-*^11^C-Me-NB1 [295, 300], which has recently been translated to humans, as well as the radiofluorinated analogs, *(R)-*^18^F-OF-Me-NB1 [292, 298], and *(R)/(S)-*^18^F-OF-NB1 [293, 296], which have recently been advanced to non-human primate studies and exhibited favorable performance characteristics, providing effective tools for the non-invasive quantification of GluN2B-carrying NMDA receptors.

## Cardiac sympathetic denervation

Cardiac sympathetic denervation in PD patients was first observed more than two decades ago, when Goldstein et al. used PET imaging to visualize the significant loss of adrenergic nerve terminals in the left ventricle [301]. Since then, numerous studies have corroborated the loss of adrenergic neurons in the myocardium of PD patients. Although autonomic function is impaired in various organs, adrenergic denervation is particularly pronounced in the heart [207]. Indeed, some PD patients present with evidence of cardiac adrenergic denervation early in the disease course. Thus, in a review by Sakakibara et al., the authors concluded that cardiac sympathetic imaging with ^123^I-metaiodobenzylguanidine (MIBG) could harbor potential for the diagnosis of pre-motor PD, in patients presenting with (non-specific) symptoms such as autonomic dysfunction, mild memory impairments, sleep disorder, visual hallucinations and depression/anxiety [302]. In addition to this potential diagnostic use, the detection of autonomic dysfunction may also have prognostic value: Autonomic dysfunction early in the course of symptomatic PD may presage a fast rate of progression [303, 304]. Mechanistic studies to explain these findings are needed. Of note, the norepinephrine derivative, droxidopa, constitutes a prodrug for the management of neurogenic orthostatic hypotension in PD patients, indicating that adrenergic signaling is a valid target for the treatment of peripheral symptoms in PD [305].

Cardiac imaging with ^123^I-MIBG can also be used to discriminate PD from MSA and progressive supranuclear palsy because the latter two conditions show only modest reduction in cardiac ^123^I-MIBG uptake, and greater sympathetic ganglion dysfunction [306, 307]. Prospective clinical trials that are adequately powered are warranted to accurately assess the diagnostic and prognostic value of cardiac sympathetic imaging in early PD as well as to identify ideal subpopulations for cardiac ^123^I-MIBG-based differential diagnosis of parkinsonism.

## Concluding remarks

Notwithstanding the significant progress in disease management, PD remains a major source of global disability, with no current cure. The main challenge in contemporary drug discovery is the identification of disease-modifying agents. Attempts to target αS are particularly promising, given the established role of αS across all stages of disease pathology. Therapeutic efficacy of αS-directed antibodies in the pipeline could potentially be monitored with a clinically validated αS-targeted probe, which holds promise to deliver an improved disease prognosis, however, their clinical development is still at an early stage and larger clinical trials that are supported by αS-specific and selective PET ligands with appropriate in vivo performance characteristics are currently pending. Beyond αS, molecular imaging with PET and SPECT has been established for the assessment of nigrostriatal loss, particularly with probes that are substrates of the dopamine transporter and serve to quantify dopaminergic nerve loss in clinical routine. More exploratory imaging approaches, that are currently mainly applied for research purposes in PD, include the assessment mitochondrial dysfunction, as well as dysregulation of the serotonergic and glutamatergic systems. To assess the role of neuroinflammation in PD patients, validated TSPO-targeted probes are employed due to their capacity to detect activated microglia. While the availability of a diverse arsenal of selective imaging probes is undoubtedly helpful as a research toolbox, further studies are required to determine the true potential of these exploratory imaging approaches to support drug discovery and improve the current standard of care for PD patients. There is an urgent need for early biomarkers that support diagnosis and patient recruitment. Further, disease progression markers are needed to facilitate the evaluation of therapeutic efficacy to improve the contemporary management of PD. Translational molecular imaging holds promise to advance the diagnostic landscape and contribute to an improved clinical decision-making. Tailored therapeutic strategies, based on individual patient scans, may help to define optimal management concepts and lead to the best possible treatment for every PD patient in the future.

## Data Availability

Not applicable.

## Abbreviations

^11^C-DTBZ: ^11^C-Dihydrotetrabenazine
^11^C-PIB: ^11^C-Pittsburgh compound-B
^11^C-CFT: ^11^C-WIN 35,428
MIBG: ^123^I-Metaiodobenzylguanidine
MPP +: 1-Methyl-4-pyridinium
^18^F-FDOPA: 6-^18^F-fluoro-levodopa
αS: α-Synuclein
AMPA: Amino-3-hydroxy-5-methyl-4-isoxazolepropionic acid
^18^F-BF227: Benzoxazole BF227
COMT: Catechol ortho methyl transferase
CNS: Central nervous system
DLB: Dementia with Lewy bodies
DDC: DOPA decarboxylase
D1: Dopamine receptor type I
D2: Dopamine receptor type II
DAT: Dopamine transporter
GLP-1: Glucagon-like peptide-1
GCase: Glucocerebrosidase
GluRs: Glutamate receptors
HDAC6: Histone deacetylase 6
KA receptors: Kainate receptors
LRRK2: Leucine-rich repeat kinase 2
MC-I: Mitochondrial complex I
MAO-B: Monoamine oxidase B inhibitors
MSA: Multiple system atrophy
MPTP: *N-*Methyl-4-phenyl-1,2,3,6-tetrahydropyridine
NMDA: N-methyl-d-aspartate
PD: Parkinson’s disease
translocator protein 18 kDa, TSPO: Peripheral benzodiazepine receptor
PET: Positron emission tomography
PTMs: Post-translational modifications
SPECT: Single-photon emission computed tomography
SNAREs: Soluble *N-*ethylmaleimide-sensitive attachment protein receptors
SNpc: Substantia nigra pars compacta
TNF: Tumor necrosis factor
FDA: US Food and Drug Administration
VMAT1: Vesicular monoamine transporter 1
VMAT2: Vesicular monoamine transporter 2
